# Digital drainage system: how far can we go?

**DOI:** 10.1590/S1806-37132014000500015

**Published:** 2014

**Authors:** Altair da Silva Costa, Luiz Eduardo Villaça Leão, Jose Ernesto Succi, Erika Rymkiewicz, Juliana Folador, Thamara Kazantzis

**Affiliations:** Department of Thoracic Surgery, Federal University of São Paulo Paulista School of Medicine Hospital São Paulo, São Paulo, Brazil; and Professor, Department of Thoracic Surgery, ABC School of Medicine, Santo André, Brazil; Department of Thoracic Surgery, Federal University of São Paulo Paulista School of Medicine, São Paulo, Brazil; Department of Thoracic Surgery, Federal University of São Paulo Paulista School of Medicine, São Paulo, Brazil; Residency Program, Department of Thoracic Surgery, Federal University of São Paulo Paulista School of Medicine Hospital São Paulo, São Paulo, Brazil; Department of Thoracic Surgery, Federal University of São Paulo Paulista School of Medicine Hospital São Paulo, São Paulo, Brazil; Department of Thoracic Surgery, Federal University of São Paulo Paulista School of Medicine Hospital São Paulo, São Paulo, Brazil

## To the Editor:

Most patients who have lung surgery require drainage of the pleural space to remove pleural fluid and air in the postoperative period and to allow adequate expansion of the remaining lung. Prolonged air leak is an expected complication in approximately 10% of patients.^(^ 1 ^)^ It is necessary that the health care team be qualified to manage the drainage system adequately. The measurement or grading of air leaks is still subjective and depends on the level of experience of professionals in quantifying them. Therefore, interpretation of air leaks is related to observer variability.^(^ 1 ^,^ 2 ^)^ Because it is subjective, there is interobserver disagreement, even among experienced observers. When uncertainty persists, the patient remains hospitalized for at least another day or period.

Since digital thoracic drainage systems became available, patients have had some advantages at their disposal.^(^ 2 ^,^ 3 ^)^ This type of system is portable and is powered by a rechargeable battery with a run time of 12 h. It has alarms and alerts for various situations, such as tube occlusion, system disconnection, suction failure, etc. Because it is a completely closed system, the fluid has no contact with the outside environment, and this provides improved biosafety for the health care team and patients themselves. Another advantage regards air leaks, since the system minimizes interobserver differences. Air leaks are measured objectively, in mL/min, and can also be seen in chart form. The suction pressure is regulated inside the device itself, which operates independently from the hospital vacuum system. When the air leak is less than 40 mL/min in the preceding 6 h, which can be seen in the chart on the device screen, the tube can be removed.^(^ 2 ^-^ 4 ^)^ The amount of liquid is measured traditionally into a graded container. Digital drainage systems are well tolerated by patients and provide greater safety and mobility. They can reduce the duration of hospital stay and its costs, since the tube is removed earlier.^(^ 2 ^,^ 4 ^,^ 5 ^)^ We report the use of a digital drainage system in a severely ill patient undergoing lung resection.

**Figure 1:**
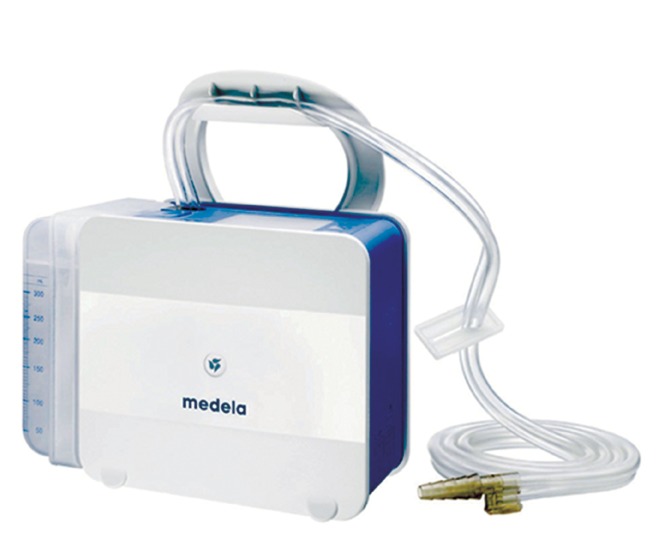
Digital thoracic drainage system.

The device was used in a severely ill elderly male with hypertension, diabetes, arteriosclerosis, COPD, and lung cancer. At the time, the patient had bloody sputum with some episodes of hemoptysis and a mass in the middle lobe. After lung resection, he remained on mechanical ventilation for 5 days, under intensive care. The patient also had an air leak from the tube, and, from postoperative day 2 onward, he was monitored with the Thopaz^(r)^ digital system (Medela, Baar, Switzerland; Figure 1).

**Figure 2:**
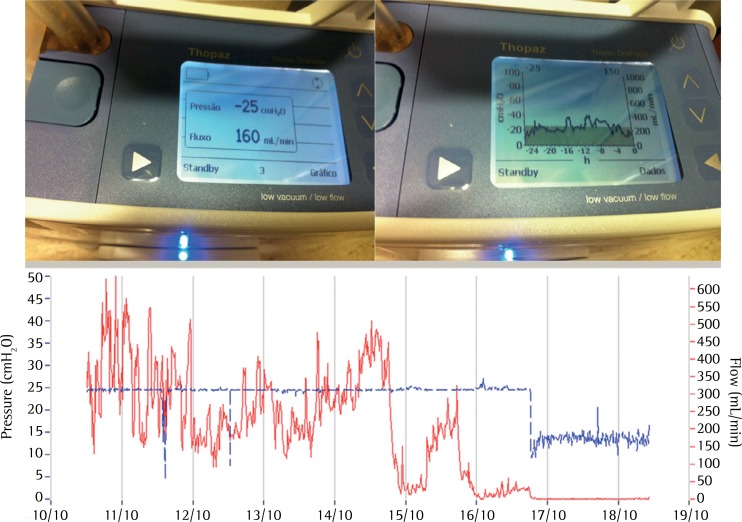
Photos of the device showing the data and graph, and, just below. the chart on the computer. In red, graphical representation of air loss (flow in mL/min); in blue, the pressure if suction is used (in cmH2O). Both representations versus time in days.

On the system screen, we can obtain information about what is currently happening, with the air leak status and the suction pressure used being displayed. In the chart, we have information about what happened in the preceding 24 h (Figure 2). Therefore, during medical or nursing rounds, we can consult this information and improve the decision-making process. In addition, the information can be exported to a computer with the ThopEasy software (Medela). We thus obtain more parameters, such as duration of drainage, with the date and the start and end time of system use, as well as minimum and maximum values for suction and air leaks (Figure 2). Suction can be quantified in various units; we chose cmH_2_O. In addition, air leaks can be measured on a scale ranging from 100 to 2,000 mL. In our patient, for instance, the tube remained in place for 7 days, and the maximum air leak was 637 mL/min (Figure 2).

The system used has functionality and simplicity and offers new standards for thoracic drainage. It allows early mobilization of patients, even for those on continuous suction, which is difficult to accomplish with the traditional water-seal system under suction.^(^ 5 ^,^ 6 ^)^ It simplifies nursing care because of improved safety and provides objective data about air leaks. Among the disadvantages are the need for training health professionals in handling the system and the "*custo Brasil*" (Brazil cost) to import it.

Although the system is routinely used in health facilities worldwide,^(^ 2 ^,^ 4 ^,^ 5 ^)^ there is still uncertainty as to which patients would benefit from its use. We need to determine where this digital system would make a difference, and, therefore, we believe that it should also be evaluated and studied in Brazil.

Note: The authors declare that there are no conflicts of interest between them and the product manufacturer or any company that is related to the product.
