# Supplementary material for: Digital drainage system: how far can we go?
Source: J Bras Pneumol. 2014 Sep-Oct;40(5):579–81. doi: 10.1590/S1806-37132014000500015 (PMC4263340; doi:10.1590/S1806-37132014000500015)
Supplement: Translation

# Translation: Sistema de drenagem digital: até onde podemos chegar?

**Authors:** Altair da Silva Costa, Luiz Eduardo Villaça Leão, Jose Ernesto Succi, Erika Rymkiewicz, Juliana Folador, Thamara Kazantzis

**Affiliations:** Disciplina de Cirurgia Torácica, Hospital São Paulo, Escola Paulista de Medicina, Universidade Federal de São Paulo, São Paulo, SP, Brasil; e Professor, Disciplina de Cirurgia Torácica, Faculdade de Medicina do ABC, Santo André, SP, Brasil; Disciplina de Cirurgia torácica, Escola Paulista de Medicina, Universidade Federal de São Paulo, São Paulo, SP, Brasil; Disciplina de Cirurgia Torácica, Escola Paulista de Medicina, Universidade Federal de São Paulo, São Paulo, SP, Brasil; Residência Médica, Disciplina de Cirurgia Torácica, Hospital São Paulo, Escola Paulista de Medicina, Universidade Federal de São Paulo, São Paulo, SP, Brasil; Disciplina de Cirurgia Torácica, Hospital São Paulo, Escola Paulista de Medicina, Universidade Federal de São Paulo, São Paulo, SP, Brasil; Disciplina de Cirurgia Torácica, Hospital São Paulo, Escola Paulista de Medicina, Universidade Federal de São Paulo, São Paulo, SP, Brasil

## Ao Editor:

A maioria das cirurgias pulmonares necessita da drenagem do espaço pleural para eliminar o líquido e o ar pleural no pós-operatório e possibilitar a expansão adequada do pulmão remanescente. A perda aérea prolongada é uma complicação esperada em aproximadamente 10% dos pacientes.^(^ 1 ^)^ A equipe precisa ser capacitada para o manejo adequado do sistema de drenagem. A medição ou a classificação dos vazamentos de ar ainda é subjetiva e depende da experiência do profissional para quantificá-los. Portanto, a interpretação da perda aérea está relacionada à variabilidade do observador.^(^ 1 ^,^ 2 ^)^ Por ser subjetiva, discordâncias entre observadores, mesmo que experientes, ocorrem. Quando persiste a duvida, o paciente permanece pelo menos mais um dia ou período internado.

Desde que os sistemas de drenagem torácica digitais surgiram, os pacientes têm a sua disposição algumas vantagens.^(^ 2 ^,^ 3 ^)^ É portátil, com autonomia para 12 h através de uma bateria recarregável. Dispõe de alarmes e alertas para diversas situações, como dreno obstruído, sistema desconectado, falha na aspiração, entre outras. Por ser um sistema completamente fechado, não existe contato do líquido com o meio externo, o que acrescenta uma enorme biossegurança à equipe e ao próprio paciente. Outra vantagem é justamente na perda aérea, pois o sistema minimiza as diferenças entre observadores. O vazamento de ar é medido de maneira objetiva, em mL/min, e também pode ser visto em forma de gráfico. A pressão de aspiração é regulada no próprio aparelho, independentemente da rede de vácuo do hospital. Quando houver uma perda aérea menor que 40 mL/min nas últimas 6 h, o que pode ser observado no gráfico no próprio aparelho, o dreno pode ser retirado. ^(^ 2 ^-^ 4 ^)^ O montante líquido é computado de forma tradicional através do recipiente graduado. O sistema digital é bem tolerado pelos pacientes, oferece mais segurança e mobilidade. Pode reduzir o período de internação e custos pois o dreno é retirado em menor tempo.^(^ 2 ^,^ 4 ^,^ 5 ^)^ Relatamos o uso de um sistema de drenagem digital em um paciente grave submetido à ressecção pulmonar.

O dispositivo foi utilizado em um paciente idoso grave, hipertenso, diabético e portador de arteriosclerose, DPOC e neoplasia pulmonar. Apresentava, na época, escarro hemóptico com alguns episódios de hemoptise e uma massa em lobo médio. Após a ressecção pulmonar, permaneceu em ventilação mecânica por cinco dias, em terapia intensiva. Também apresentou perda aérea pelo dreno e a partir do segundo dia pós-operatório foi monitorizado pelo sistema digital Thopaz^(r)^ (Medela, Baar, Suíça; Figura 1). 

**Figura 1:** Sistema digital para drenagem torácica.

Através do visor, podemos obter informações do que ocorre no momento, com a indicação da perda aérea e da pressão de aspiração utilizada. Pelo gráfico, temos as informações do que ocorreu nas últimas 24 h (Figura 2). Assim, durante as visitas médicas ou de enfermagem, podemos consultar essas informações e tomar decisões melhores. Além disso, as informações podem ser exportadas para um computador através do programa ThopEasy (Medela). Assim, obtemos mais parâmetros, como o tempo de drenagem, com a data e a hora inicial e final de uso do sistema, valor máximo e valor mínimo de aspiração e de perda aérea (Figura 2). A aspiração pode ser regulada em diversas unidades; nós optamos por cmH_2_O. A perda aérea também pode ser medida em uma escala, que varia de 100 a 2.000 mL. Nosso paciente, por exemplo, ficou com o dreno por sete dias, com uma perda aérea máxima de 637 mL/min (Figura 2).

**Figura 2:** Acima Dados e gráfico no próprio dispositivo. Abaixo, os gráficos no computador. Em vermelho, representação gráfica da perda aérea (fluxo em mL/min); em azul, a pressão se aspiração utilizada (em cmH2O); ambas em relação ao tempo, em dias.

O sistema utilizado apresenta funcionalidade e simplicidade e oferece novos padrões para a drenagem torácica. Possibilita a mobilização precoce do paciente mesmo em aspiração continua, o que é difícil com o sistema tradicional de selo d'água sob aspiração.^(^ 5 ^,^ 6 ^)^ Simplifica os cuidados de enfermagem com o aumento da segurança e fornece dados objetivos sobre a perda aérea. Como desvantagens, citamos ser necessário o treinamento dos profissionais de saúde para o manuseio do sistema e o "custo Brasil" para sua importação. 

Apesar de o sistema ser utilizado na rotina em serviços no mundo,^(^ 2 ^,^ 4 ^,^ 5 ^)^ ainda restam dúvidas sobre quais pacientes seriam beneficiados por seu uso. Precisamos determinar onde o sistema digital faria a diferença e, portanto, acreditamos que também esse sistema deva ser avaliado e estudado em nosso meio.

Observação: Os autores declaram que não há conflito de interesse com o fabricante ou qualquer empresa relacionada ao produto.
